# *WTAP* gene variants and susceptibility to ovarian endometriosis in a Chinese population

**DOI:** 10.3389/fgene.2023.1276099

**Published:** 2023-10-13

**Authors:** Zixian Wan, Lu Ye, Guange Chen, Chaoyi Xiong, Zhenbo OuYang, Liangzhi Wu, Jing He, Ping Duan, Youkun Jie, Qiushi Zhang, Wenfeng Hua

**Affiliations:** ^1^ Research Institute for Maternal and Child Health, Guangdong Second Provincial General Hospital, Guangzhou, Guangdong, China; ^2^ Department of Gynecology, Guangdong Second Provincial General Hospital, Guangzhou, Guangdong, China; ^3^ Department of Pathology, Jiangxi Maternal and Child Health Hospital, Nanchang, Jiangxi, China; ^4^ Department of Obstetrics and Gynecology, The Second Affiliated Hospital and Yuying Children’s Hospital of Wenzhou Medical University, Wenzhou, Zhejiang, China; ^5^ Department of Pediatric Surgery, Guangzhou Institute of Pediatrics, Guangzhou Women and Children’s Medical Center, Guangzhou Medical University, Guangzhou, Guangdong, China

**Keywords:** endometriosis, infertility, gene polymorphism, WTAP, Chinese population

## Abstract

**Background:** Endometriosis is a common chronic gynecologic disorder with a significant negative impact on women’s health. Wilms tumor 1-associated protein (WTAP) is a vital component of the RNA methyltransferase complex for N^6^-methyladenosine modification and plays a critical role in various human diseases. However, whether single nucleotide polymorphisms (SNPs) of the WTAP gene predispose to endometriosis risk remains to be investigated.

**Methods:** We genotyped three WTAP polymorphisms in 473 ovarian endometriosis patients and 459 control participants using the Agena Bioscience MassArray iPLEX platform. The logistic regression models were utilized to assess the associations between WTAP SNPs and the risk of ovarian endometriosis.

**Results:** In the single-locus analyses, we found that the rs1853259 G variant genotypes significantly increased, while the rs7766006 T variant genotypes significantly decreased the association with ovarian endometriosis risk. Combined analysis indicated that individuals with two unfavorable genotypes showed significantly higher ovarian endometriosis risk (adjusted OR = 1.71 [1.23–2.37], *p* = 0.001) than those with zero risk genotypes. In the stratified analysis, the risk effect of the rs1853259 AG/GG and rs7766006 GG genotypes was evident in subgroups of age ≤30, gravidity≤1, parity≤1, rASRM stage I, and the rs7766006 GG genotype was associated with worse risk (adjusted OR = 1.64 [1.08–2.48], *p* = 0.021) in the patients with rASRM stage II + III + IV. The haplotype analysis indicated that individuals with GGG haplotypes had a higher risk of ovarian endometriosis than wild-type AGG haplotype carriers. Moreover, false positive report probability and Bayesian false discovery probability analysis validated the reliability of the significant results. The quantitative expression trait loci analysis revealed that rs1853259 and rs7766006 were correlated with the expression levels of WTAP.

**Conclusion:** Our findings demonstrated that WTAP polymorphisms were associated with susceptibility to ovarian endometriosis among Chinese women.

## 1 Introduction

Endometriosis, characterized by severe menstrual problems and pelvic pain, is a common gynecological disorder with an estrogen-dependent chronic inflammatory process (Taylor et al., 2021). About 10% of women of reproductive age globally and is strongly linked with infertility (Giudice and Kao, 2004; Zondervan et al., 2020). Although the etiology of endometriosis remains unclear, the interplay of multiple genetic and environmental factors was a significant contributor (Koninckx et al., 2019; Mear et al., 2020).

In two extensive twin studies, the concordance rate of endometriosis among monozygotic twins is approximately 50% (Treloar et al., 1999; Saha et al., 2015), suggesting that genetic factors play a vital role in the development of endometriosis. Therefore, to better understand the genetic risk variants of endometriosis, it is essential to identify genetic markers that affect the disease pathogenesis.

Wilms tumor 1-associated protein (WTAP) is a ubiquitously expressed nuclear protein whose transcription is regulated by Wilms tumor protein 1(WT-1) (Little et al., 2000). Recently, WTAP has been identified as a regulatory subunit in the RNA N^6^-methyladenosine (m6A) methyltransferase complex and plays a vital role in epi-transcriptomic regulation of RNA metabolism, independent of WT-1 (Ping et al., 2014; Huang et al., 2022). Furthermore, studies have shown that WTAP plays a critical role in the regulation of many physiological and pathological processes, such as alternative splicing (Horiuchi et al., 2021), cell cycle regulation (Horiuchi et al., 2006), cell proliferation (Villa et al., 2021), embryo development (Fukusumi et al., 2008; Hao et al., 2021), and tumorigenesis (Chen et al., 2020; Han et al., 2021). In addition, studies showed that genetic variants of WTAP might alter the gene’s ability to fulfill its biological functions (Ma et al., 2020; Zhuo et al., 2020; He et al., 2021; Tang et al., 2021). However, whether WTAP genetic variations also affect endometriosis risk is still unclear. With this in mind, we conducted a case-control study investigating the association between functional SNPs in the WTAP gene and endometriosis risk among Chinese women.

## 2 Methods

### 2.1 Study subjects

This case-control study included 473 cases and 459 controls recruited from Jiangxi Provincial Maternal and Child Health Hospital between January 2015 and June 2022. All the patients were diagnosed with ovarian endometriosis and staged by histologically examining tissues from biopsy or resected specimens according to the revised American Society for Reproductive Medicine classification (rASRM). Expert pathologists re-reviewed all ovarian endometriosis tissue slides. The participants in the control group were healthy female volunteers without a family history of endometriosis and malignant neoplasm. The cases were paraffin-embedded ovarian endometriosis tissue samples from the Department of Pathology archives at Jiangxi Provincial Maternal and Child Health Hospital. The controls were peripheral blood samples of age-matched healthy controls undergoing health examinations in the same hospital simultaneously.

### 2.2 SNPs selection and genotyping

The three potentially functional WTAP gene SNPs (rs1853259 A>G, rs7766006 G>T, and rs9457712 G>A) were selected from previous studies (Ma et al., 2020; Zhuo et al., 2020; He et al., 2021; Tang et al., 2021). Three SNPs with minor allele frequencies (MAFs) > 0.05 among the Chinese population and the detailed information of these SNPs, as shown in Supplementary Table S1. No significant linkage disequilibrium (LD) (*R* ^2^ < 0.8) among these three SNPs, as previously stated (Zhuo et al., 2020). The paraffin-embedded tissue samples and peripheral blood samples were used to extract genomic DNA by the QIAamp DNA FFPE Tissue Kit (Qiagen, Valencia, CA) and Genome TIANGEN Blood DNA Extraction Kit (TianGen Biotech, Beijing) according to the manufacturer’s protocol, respectively. Subsequently, using the Agena Bioscience MassArray iPLEX platform, genotyped the DNA samples performed by CapitalBio Technology (Beijing) based on MALDI-TOF (matrix-assisted laser desorption/ionization-time-of-flight) mass spectrometry—the PCR primers for running the assay on the MassArray system, as shown in Supplementary Table S2. The genotyping completion rate for all SNPs was more than 95%.

### 2.3 Statistical analysis

A goodness-of-fit Chi-square test was used to assess the Hardy-Weinberg equilibrium (HWE) of WTAP gene SNPs in controls. Analyze the demographic and clinical characteristics difference between the cases and controls using Student’s t-test (for continuous variables) and the Chi-square test (for categorical variables). Unconditional logistic regression analysis was used to calculate crude and adjusted odds ratios (ORs) along with 95% confidence intervals (CIs). Associations between the genotypes and risk of ovarian endometriosis among subgroups of age, gravidity, parity, and rASRM stage were further evaluated by stratification analysis. To assess the haplotype frequency and its effect on ovarian endometriosis risk by using a logistic regression model. Using the Genotype-Tissue Expression (GTEx) portal website (http://www.gtexportal.org/home/), the quantitative expression trait locus (eQTL) analysis was adopted to predict the potential associations between the SNPs and expression levels of the WTAP gene.

Additionally, the false positive report probability (FPRP) and Bayesian false discovery probability (BFDP) were used to evaluate the robustness of significant findings, as described elsewhere (Wacholder et al., 2004; Wakefield, 2007). First, FPRP tests the probability of no true association between the genetic variant and disease risk. The magnitude of the FPRP value is determined by statistical power, observed *p*-value, and prior probability. SAS software calculated the statistical power and FPRP values with an OR of 1.50 (risk effects) or 0.67 (protective effects) under the range of prior probabilities from 0.25 to 0.01. As previously suggested, a cutoff value of 0.2 is the threshold of FPRP. Any finding with an FPRP value of <0.2 is considered noteworthy; second, utilized BFDP to clarify the noteworthiness of the significant results with a sound methodological basis. It describes noteworthiness using the cost of a false discovery and a false non-discovery. According to the literature, a cutoff value of 0.8 as the BFDP threshold derived from the assumption that a false non-discovery is four times as costly as a false discovery. The prior probabilities applied in the BFDP calculation were the same as FPRP’s, and any finding with a BFDP value of <0.8 is considered noteworthy. All statistical analyses were conducted using SAS 9.4 (SAS Institute Inc., Cary, NC), except the BFDP value using the Excel spreadsheet released by Wakefield et al. (Wakefield, 2007). P < 0.05 was considered statistically significant.

## 3 Results

### 3.1 Characteristics of the study population

Table 1 displays several clinical characteristics of 473 ovarian endometriosis patients and 459 control participants. The mean age of the patients and controls were 31.97 ± 6.06 and 31.14 ± 7.07, respectively. Both groups had a majority of participants under 35 (69.34% and 76.91%, respectively). There were no statistically significant differences in age, gravidity, and parity between the patients and controls.

**TABLE 1:** Frequency distribution of selected variables for ovarian endometriosis cases and controls.

Characteristic	Cases (N = 473)	Controls (N = 459)	p
Age (year, Mean ± SD)	31.97 ± 6.06	31.14 ± 7.07	0.054^a^
<35	328 (69.34%)	353 (76.91%)	
≥35	145 (30.66%)	106 (23.09%)	
Gravidity			0.051^b^
≤1	423 (89.4%)	391 (85.2%)	
>1	50 (10.6%)	68 (14.8%%)	
Parity			0.101^b^
≤1	430 (90.91%)	402 (87.58%)	
>1	43 (9.09%)	57 (12.42%)	
rASRM stage^c^			
I	349 (73.78%)		
II	104 (21.99%)		
III	15 (3.17%)		
IV	5 (1.06%)		

^a^ Student’s t-test for distributions between cases and controls.

^b^ χ2 test for distributions between cases and controls.

^c^ rASRM: the revised American Society for Reproductive Medicine staging system.

According to the rASRM classification, 349 (73.78%) women had endometriosis stage I (minimal), 104 (21.99%) stage II (mild), 15 (3.17%) stage III (moderate), 5 (1.06%) stage IV (severe).

### 3.2 Associations between WTAP polymorphisms and the risk of ovarian endometriosis

Association analysis of WTAP gene SNPs and ovarian endometriosis risk is shown in Table 2. All the observed genotype frequencies for the three selected SNPs agreed with the HWE in the controls (*p* = 0.847 for rs1853259, *p* = 0.633 for rs776006, and *p* = 0.932 for rs9457712). In the single-locus analysis, the rs1853259 G variant genotypes were significantly associated with increased ovarian endometriosis risk (AG vs AA: adjusted odds ratio [AOR] = 1.34, 95% confidence interval [CI] = 1.01–1.79, *p* = 0.044; GG vs AA: AOR = 1.61, 95% CI = 1.09–2.40, *p* = 0.018; p_trend_ = 0.032; additive model: AOR = 1.29, 95% CI = 1.06–1.56, *p* = 0.01; dominant model: AOR = 1.40, 95% CI = 1.07–1.84,*p* = 0.014). In contrast, reduced risk of ovarian endometriosis was observed when examining the association with the rs776006 T variant genotypes (GT vs GG: AOR = 0.73, 95% CI = 0.54–0.98, *p* = 0.035; TT vs GG: AOR = 0.61, 95% CI = 0.42–0.89, *p* = 0.011; p_trend_ = 0.022; additive model: AOR = 0.77, 95% CI = 0.64–0.93, *p* = 0.007; dominant model: AOR = 0.69, 95% CI = 0.52–0.92, *p* = 0.01). However, there were no significant associations between the rs9457712 variant alleles and ovarian endometriosis risk in this study. Subsequently, the rs1853259 AG/GG, rs7766006 GG, and rs9457712 AA were defined as risk genotypes based on their ORs to test their combined effect on the risk of ovarian endometriosis. Individuals with one risk genotype showed a 1.38-fold increase (95% CI = 1.001–1.89, *p* = 0.05), and those with two risk genotypes showed a 1.71-fold increase in the risk of developing ovarian endometriosis (95% CI = 1.23–2.37, *p* = 0.001; p_trend_ = 0.008) when compared with those without risk genotype.

**TABLE 2:** Association between WTAP gene polymorphisms and ovarian endometriosis susceptibility.

Genotype	Cases (N = 473)	Controls (N = 459)	Crude OR (95% CI)	p^a^	Adjusted OR (95% CI)	p^b^
WTAP rs1853259 A>G, HWE = 0.847						
AA	153 (32.34%)	181 (39.43%)	1.00		1.00	
AG	235 (49.68%)	216 (47.06%)	1.29 (0.97–1.71)	0.081	1.34(1.01–1.79)	0.044
GG	85 (17.97%)	62 (13.50%)	1.62 (1.10–2.40)	0.016	1.61 (1.09–2.40)	0.018
			p_trend_ = 0.039		p_trend_ = 0.032	
Additive			1.28 (1.06–1.54)	0.011^c^	1.29 (1.06–1.56)	0.010^c^
Dominant			1.36 (1.04–1.78)	0.024	1.40 (1.07–1.84)	0.014
Recessive			1.40 (0.98–2.01)	0.062	1.36 (0.95–1.96)	0.093
WTAP rs7766006 G>T, HWE = 0.633						
GG	175 (37.00%)	132 (28.76%)	1.00		1.00	
GT	220 (46.51%)	233 (50.76%)	0.71 (0.53–0.95)	0.023	0.73 (0.54–0.98)	0.035
TT	78 (16.49%)	94 (20.48%)	0.63 (0.43–0.91)	0.015	0.61 (0.42–0.89)	0.011
			p_trend_ = 0.022		p_trend_ = 0.022	
Additive			0.78 (0.65–0.94)	0.008^c^	0.77 (0.64–0.93)	0.007^c^
Dominant			0.69 (0.52–0.90)	0.008	0.69 (0.52–0.92)	0.010
Recessive			0.77 (0.55–1.07)	0.117	0.74 (0.53–1.03)	0.076
WTAP rs9457712 G>A, HWE = 0.932						
GG	333 (70.40%)	316 (68.85%)	1.00		1.00	
GA	120 (25.37%)	130 (28.32%)	0.88 (0.65–1.17)	0.374	0.88 (0.65–1.18)	0.393
AA	20 (4.23%)	13 (2.83%)	1.46 (0.72–3.06)	0.300	1.47 (0.72–3.09)	0.300
			p_trend_ = 0.350		p_trend_ = 0.363	
Additive			1.00 (0.79–1.26)	0.964^c^	1.00 (0.79–1.27)	0.986^c^
Dominant			0.93 (0.70–1.23)	0.605	0.93 (0.70–1.24)	0.629
Recessive			1.52 (0.75–3.16)	0.252	1.52 (0.75–3.19)	0.254
The combined effect of risk genotypes						
0	132 (27.91%)	167 (36.38%)	1.00		1.00	
1	167 (35.30%)	161 (35.08%)	1.31 (0.96–1.80)	0.090	1.38 (1.001–1.89)	0.050
2	174 (36.79%)	131 (28.54%)	1.68 (1.22–2.32)	0.002	1.71 (1.23–2.37)	0.001
			p_trend_ = 0.007		p_trend_ = 0.008	

^a^ χ2test for genotype distributions between cases and controls.

^b^ Adjusted for age, abortion, and parity.

^c^ Genotypes analyzed by the Cochran-Armitage test.

^d^ Risk genotypes were rs1853259AG/GG, rs7766006 GG, and rs9457712AA.

P_trend_ Analyzed by the Cochran-Armitage test.

### 3.3 Stratification analysis

Stratification analysis based on age, gravidity, parity, and rASRM stage was further performed. As shown in Table 3, the rs1853259 AG/GG genotype was associated with increased ovarian endometriosis risk in groups of age ≤30 (AOR = 1.57, 95% CI = 1.07–2.31, *p* = 0.021), gravidity≤1 (AOR = 1.35, 95% CI = 1.01–1.80, *p* = 0.042), parity≤1 (AOR = 1.35, 95% CI = 1.02–1.80, *p* = 0.039), and rASRM stage I (AOR = 1.45, 95% CI = 1.08–1.95, *p* = 0.015) when compared with the AA genotype. Similarly, individuals carrying the rs7766006 GG genotype had a significantly increased risk of ovarian endometriosis in the groups of age ≤30 (AOR = 1.51, 95% CI = 1.01–2.26, *p* = 0.045), gravidity≤1 (AOR = 1.43, 95% CI = 1.06–1.93, *p* = 0.019), parity≤1 (AOR = 1.46, 95% CI = 1.09–1.96, *p* = 0.012), rASRM stage I (AOR = 1.38, 95% CI = 1.02–1.86, *p* = 0.037), and rASRM stage II + III + IV (AOR = 1.64, 95% CI = 1.08–2.48, *p* = 0.021) when compared with the GT/TT genotype. However, no significant association was found between rs9457712 and ovarian endometriosis risk in any subgroups.

**TABLE 3:** Stratification analysis for the association between WTAP gene genotypes and ovarian endometriosis risk.

Variables	rs1853259 A>G (cases/Controls)		AOR (95% CI)	p^a^	rs7766006 G>T (cases/Controls)		AOR (95% CI)	p^a^	rs9457712 G>A (cases/Controls)		AOR (95% CI)	p^a^
	AG/GG	AA			GG	GT/TT			AA	GG + GA		
Age												
≤30	184/223	69/107	1.57 (1.07–2.31)	0.021	74/65	138/184	1.51 (1.01–2.26)	0.045	8/7	204/242	1.30 (0.46–3.76)	0.623
31–40	163/132	65/61	1.27 (0.81–2.01)	0.297	82/52	125/110	1.46 (0.93–2.30)	0.104	10/6	197/156	1.54 (0.51–5.19)	0.458
>40	41/42	19/13	0.68 (0.27–1.65)	0.395	19/15	35/33	1.12 (0.48–2.63)	0.798	2/0	52/48	NA	0.988
Gravidity												
≤1	313/256	152/168	1.35 (1.01–1.80)	0.042	171/122	294/302	1.43 (1.06–1.93)	0.019	20/13	455/411	1.76 (0.82–4.00)	0.159
>1	7/22	1/13	0.94 (0.38–2.34)	0.891	4/10	4/25	1.76 (0.76–4.10)	0.188	0/0	8/35	0.52 (0.03–4.35)	0.584
Parity												
≤1	293/245	137/157	1.35 (1.02–1.80)	0.039	157/114	273/288	1.46 (1.09–1.96)	0.012	19/11	411/391	1.63 (0.78–3.59)	0.204
>1	27/33	16/24	0.91 (0.32–2.54)	0.852	18/18	25/39	1.20 (0.43–3.30)	0.731	1/2	42/55	0.86 (0.04–13.27)	0.914
rASRM stage												
I	239/278	110/181	1.45 (1.08–1.95)	0.015	125/132	224/327	1.38 (1.02–1.86)	0.037	12/13	337/446	1.25 (0.55–2.80)	0.588
II + III + IV	81/278	43/181	1.27 (0.84–1.94)	0.261	50/132	74/327	1.64 (1.08–2.48)	0.021	8/13	116/446	2.52 (0.96–6.31)	0.050

^a^ Adjusted for age, gravidity, and parity.

### 3.4 Associations between WTAP haplotypes and ovarian endometriosis risk

Next, whether the haplotypes of the three WTAP gene SNPs are linked with ovarian endometriosis risk was also analyzed. As shown in Table 4, haplotype GGG was linked with significantly increased ovarian endometriosis risk (AOR = 1.37, 95% CI = 1.03–1.82, *p* = 0.032) compared to reference haplotype AGG.

**TABLE 4:** Association between inferred haplotypes of the WTAP gene and ovarian endometriosis risk.

rs1853259 A>G	rs7766006 G>T	rs9457712 G>A	Cases (N = 473)	Controls (N = 459)	Crude OR (95% CI)	p	Adjusted OR (95% CI)	p^a^
A	G	G	286	290	1.00			
A	G	A	45	27	1.69 (1.02–2.80)	0.041	1.66 (1.00–2.76)	0.051
A	T	G	157	189	0.84 (0.65–1.10)	0.208	0.81 (0.62–1.06)	0.116
A	T	A	53	72	0.75 (0.51–1.10)	0.142	0.73 (0.49–1.08)	0.113
**G**	**G**	**G**	173	123	1.43 (1.08–1.89)	0.014	1.37 (1.03–1.82)	0.032
G	G	A	66	57	1.17 (0.80–1.73)	0.420	1.18 (0.79–1.75)	0.420
G	T	G	166	160	1.05 (0.80–1.38)	0.715	1.06 (0.81–1.40)	0.664
G	T	A	0	0	-	-	-	-

^a^ Adjusted for age, gravidity, and parity.

### 3.5 FPRP and BFDP values for all significant associations

To examine the statistical robustness, FPRP and BFDP were conducted to judge the credibility of all statistically significant associations (*p* < 0.05). As shown in Table 5, FPRP and BFDP values were calculated using three levels of prior probabilities of 0.25, 0.1, and 0.01. At a prior probability level of 0.25, all significant associations were noteworthy in the BFDP test, and 15 of the 17 significant associations were noteworthy in the FPRP test, except for the associations of rs1853259 AG vs AA and the subgroup of age ≤30 in rs7766006 GG vs GT/TT stratification analysis. When assuming a prior probability of 0.1, only 4 of the 17 significant associations were noteworthy in both tests: the rs7766006 dominant model, the subgroup of parity ≤1 in rs1853259 GG vs GT/TT stratification analysis, the combined effect of risk genotypes (2 vs 0), and haplotype analysis (GGG vs AGG). However, when calculating at a prior probability of 0.01, no noteworthy result was observed in all significant associations via the FPRP or BFDP test.

**TABLE 5:** FPRP and BFDP values for all significant associations.

	Crude OR^a^ (95% CI)	p	Statistical power^b^	Prior probability FPRP/BFDP		
				0.25	0.1	0.01
rs1853259						
AG vs AA	1.29 (0.97–1.71)	0.081	0.851	0.222/0.646	0.461/0.846	0.904/0.984
GG vs AA	1.62 (1.10–2.40)	0.016	0.349	0.121/0.492	0.292/0.744	0.819/0.970
Dominant model	1.36 (1.04–1.78)	0.024	0.753	0.087/0.488	0.223/0.741	0.759/0.969
AG/GG vs AA						
Age ≤30	1.56 (1.07–2.29)	0.022	0.785	0.078/0.528	0.201/0.771	0.735/0.974
Gravidity ≤1	1.37 (1.03–1.83)	0.031	0.763	0.109/0.534	0.268/0.775	0.801/0.974
Parity ≤1	1.37 (1.03–1.82)	0.030	0.726	0.110/0.518	0.271/0.763	0.804/0.973
rASRM stage I	1.42 (1.06–1.90)	0.020	0.647	0.085/0.450	0.218/0.711	0.754/0.964
rs7766006						
GT vs GG	0.71 (0.53–0.95)	0.023	0.666	0.094/0.471	0.237/0.728	0.774/0.967
TT vs GG	0.63 (0.43–0.91)	0.015	0.372	0.108/0.458	0.266/0.717	0.800/0.965
Dominant model	0.69 (0.52–0.90)	0.008	0.584	0.039/0.285	0.110/0.544	0.575/0.929
GG vs GT/TT						
Age ≤30	1.52 (1.02–2.26)	0.041	0.479	0.204/0.590	0.435/0.812	0.894/0.979
Gravidity ≤1	1.41 (1.05–1.90)	0.022	0.706	0.086/0.492	0.219/0.744	0.755/0.970
Parity ≤1	1.45 (1.08–1.95)	0.012	0.586	0.058/0.415	0.156/0.680	0.670/0.959
rASRM stage I	1.38 (1.03–1.86)	0.033	0.711	0.122/0.543	0.295/0.781	0.821/0.975
rASRM stage II + III + IV	1.67 (1.10–2.53)	0.014	0.302	0.122/0.501	0.294/0.751	0.821/0.971
Combined effect of risk genotypes						
2 vs 0	1.68 (1.22–2.32)	0.002	0.270	0.022/0.188	0.062/0.409	0.423/0.884
Haplotype analysis						
GGG vs AGG	1.43 (1.08–1.89)	0.014	0.643	0.061/0.383	0.164/0.650	0.683/0.953

^a^ Crude ORs, from Tables 2, 3.

^b^ Statistical power was calculated using the number of observations, Crude OR, and *p* values.

### 3.6 Effect of SNPs on gene expression

**FIGURE 1:**
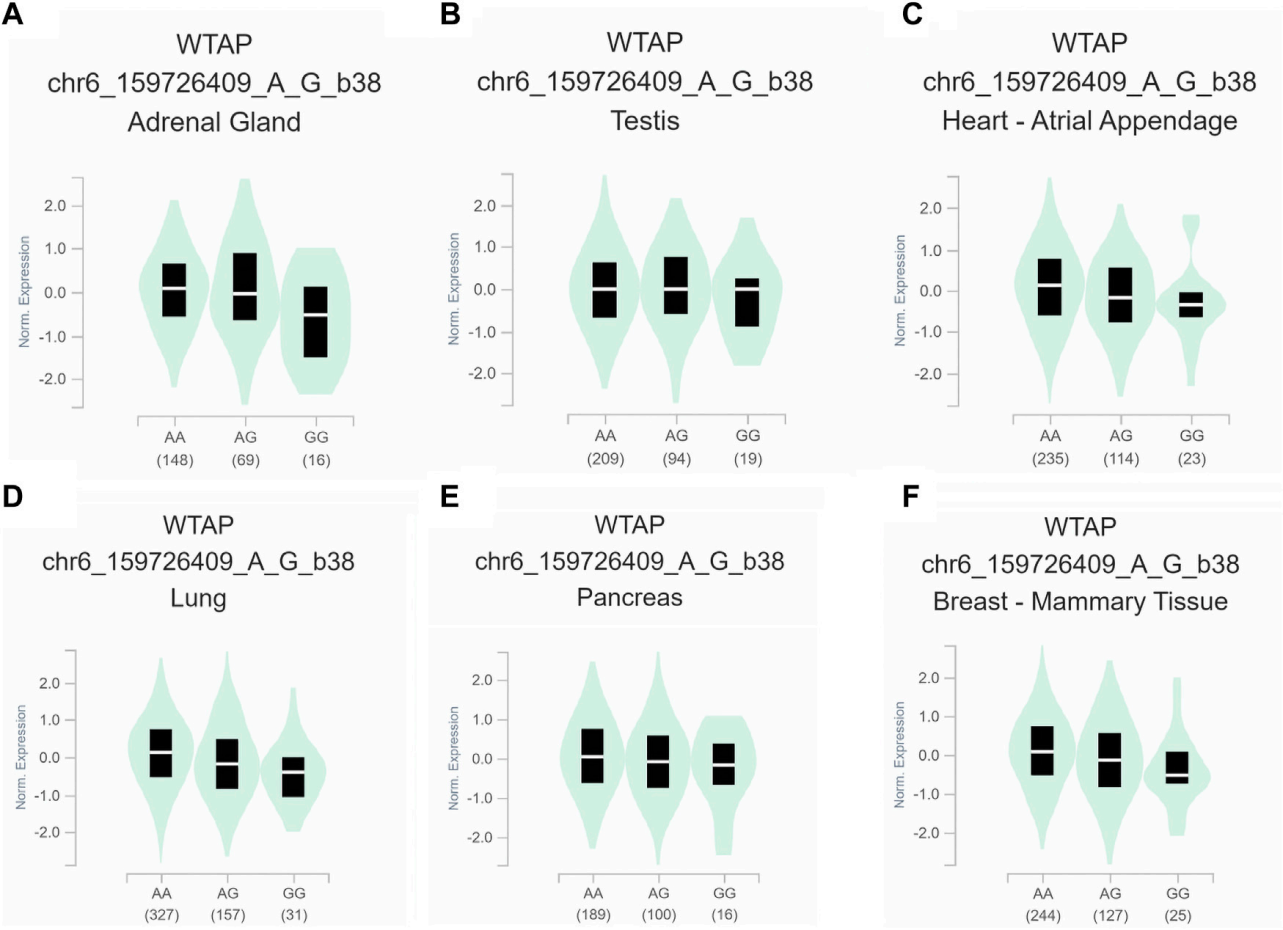
Functional relevance of rs1853259 on gene expression in GTEx database. **(A)** adrenal gland (*p* = 3.0 × 10^−5^). **(B)** testis (*p* = 3.8 × 10^−5^). **(C)** heart-atrial appendage (*p* = 4.0 × 10^−5^). **(D)** lung (*p* = 4.5 × 10^−8^). **(E)** pancreas (*p* = 6.1 × 10^−6^). **(F)** breast-mammary tissue (*p* = 3.6 × 10^−8^).

**FIGURE 2:**
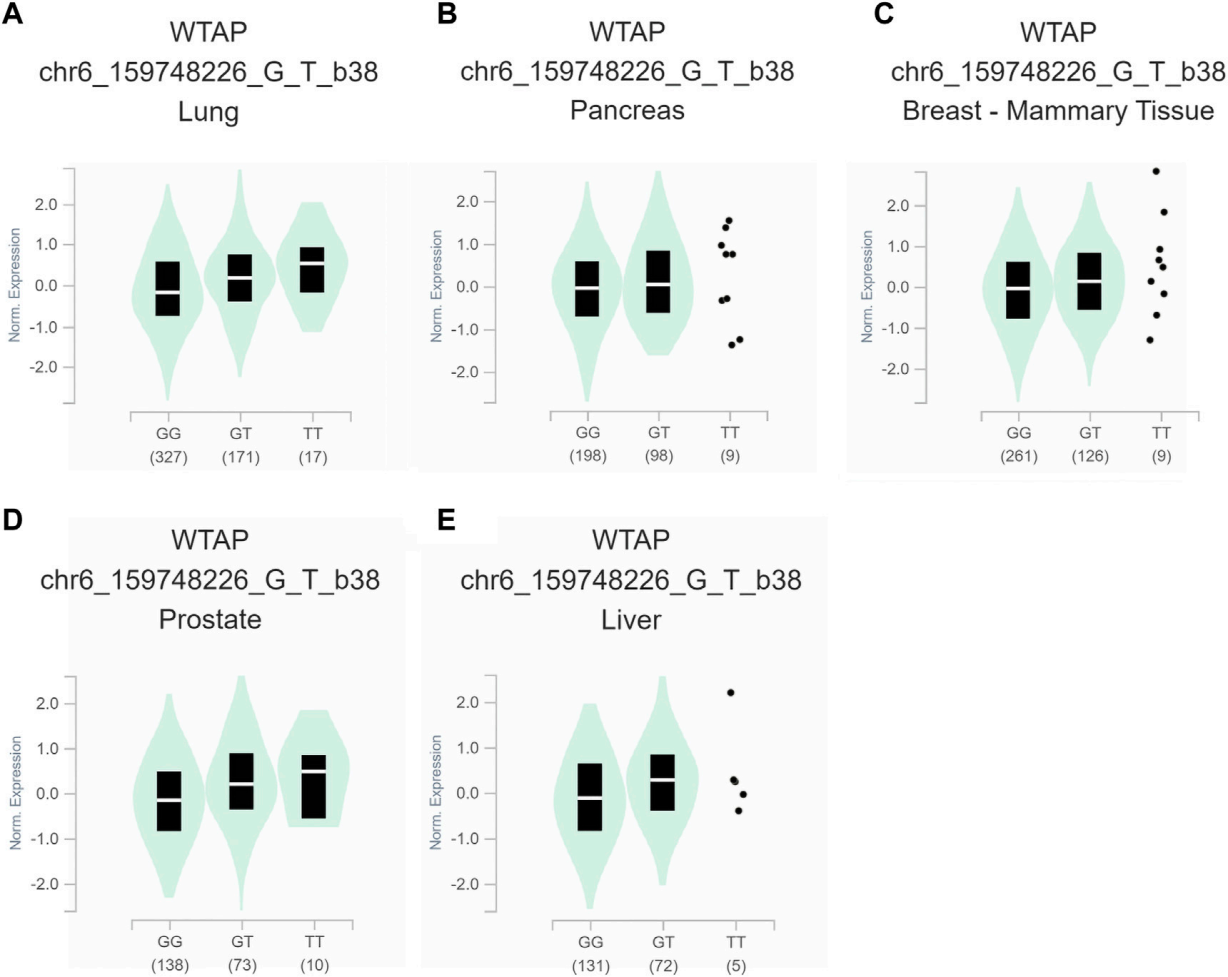
Functional relevance of rs7766006 on gene expression in GTEx database. **(A)** lung (*p* = 5.7 × 10^−6^). **(B)** pancreas (*p* = 4.3 × 10^−5^). **(C)** breast-mammary tissue (*p* = 2.4 × 10^−5^). **(D)** prostate (*p* = 3.8 × 10^−8^). **(E)** liver (*p* = 2.9 × 10^−5^).

To further assess the functional implication of the WTAP genotypes, the eQTL analysis was used to explore the effects of rs1853259 and rs7766006 on gene expression. It was found that individuals carrying the rs1853259 G genotype had significantly lower levels of WTAP expression compared to those with the rs1853259 A genotype in various organs, including the adrenal gland, testis, heart-atrial appendage, lung, pancreas, and breast-mammary tissue (Figure 1). In contrast, individuals carrying the rs7766006 T genotype had significantly higher levels of WTAP mRNA than those with the rs7766006 G genotype in different organs (Figure 2). Altogether, these findings suggest that the upregulation of WTAP in ovarian endometriosis might function as a protective effect.

## 4 Discussion

Endometriosis is a complex and distressing chronic inflammatory disease affecting about 10% of women of reproductive age. It is worth noting that the severe impact of endometriosis on infertility and its burden on the public make understanding the pathogenesis, prevention, and treatment more urgent. In recent years, the genetic component of endometriosis has received widespread attention (Sapkota et al., 2017; Gallagher et al., 2019; Adewuyi et al., 2022; Koller et al., 2023). However, critical genetic variations associated with the pathogenesis of endometriosis are still limited.

Recent emerging studies suggest that m6A modification is involved in developing and progressing endometriosis (Li et al., 2021; Wang et al., 2022; Wan et al., 2023). Jiang et al. found that most m6A regulators were significantly downregulated in eutopic and ectopic compared to the normal endometrium (Jiang et al., 2020). Similarly, Zhai et al. demonstrated that women with adenomyosis have lower m6A levels in their endometrium and myometrium than healthy individuals (Zhai et al., 2020). Notably, a recent study by Wan et al. identified that the downregulation of METTL3 expression in the uterus endometrium is a critical factor in female infertility among those with endometriosis; they proved this through conditional deletion of *Mettl3* in the reproductive tract of female mice (Wan et al., 2023). These findings suggest that the downregulation of m6A levels is closely related to the pathogenesis of endometriosis and infertility. Therefore, whether WTAP polymorphism impacts the susceptibility of endometriosis or infertility is worth further exploration.

Accumulating evidence has shown that WTAP plays a crucial role in the m6A methyltransferase complex (Ge et al., 2021; Su et al., 2022), and its knockdown leads to a significant 6.25-fold reduction in m6A transcriptome-wide (Schwartz et al., 2014). In the present study, for the first time, we found that the rs1853259 G allele and rs7766006 T allele were significantly associated with increased or decreased ovarian endometriosis risk, respectively. Based on the eQTL analysis, it is evident that the rs1853259 G allele leads to a reduction in the expression of WTAP, while the rs7766006 T allele increases the expression of WTAP (Figures 1, 2). Consistent with previous studies (Jiang et al., 2020; Zhai et al., 2020; Wan et al., 2023), our results also support that the m6A level is reduced in endometrium associated with an increased risk of ovarian endometriosis. Furthermore, stratification analysis found that the rs1853259 AG/GG and rs7766006 GG genotypes are significantly associated with increased ovarian endometriosis risk in patients younger than 30, gravidity≤1, and parity≤1 when compared to the AA genotype and GT/TT genotype, respectively. The results of eQTL analysis showed that having the rs1853259 AG/GG and rs7766006 GG genotypes were strongly associated with lower levels of WTAP. This study suggests that individuals with ovarian endometriosis and lower levels of WTAP have a higher likelihood of experiencing infertility.

Our study reinforces the idea that susceptibility variants in the genomic region of WTAP could influence ovarian endometriosis risk. However, we should note several limitations of this study. First, as a hospital-based case-control study, this study might have inherent biases due to non-representative subject selection and retrospective exposure data collection. Second, only three genotyped common polymorphisms of WTAP were present, which required uncovering more potential functional polymorphisms. Third, we should have analyzed the impact of other risk factors on endometriosis susceptibility due to the lack of information on participants. Fourth, the significant findings need additional studies to verify. Additionally, we selected the samples from one center, which may have selection bias and information bias. Finally, we also acknowledged that this study’s conclusion was limited to Chinese women.

In conclusion, our study demonstrated the impact of WTAP polymorphisms on ovarian endometriosis risk among Chinese women. Additionally, a larger sample size and different populations are required to validate this association, and functional studies are needed to unveil the underlying role of the WTAP gene SNPs in endometriosis risk.

## Data Availability

The datasets presented in this study can be found in online repositories. The names of the repository/repositories and accession number(s) can be found in the article/Supplementary Material.
