# Supplementary material for: WTAP gene variants and susceptibility to ovarian endometriosis in a Chinese population
Source: Front Genet. 2023 Oct 13;14:1276099. doi: 10.3389/fgene.2023.1276099 (PMC10603221; doi:10.3389/fgene.2023.1276099)
Supplement: Supplementary file 1 [file Table1.DOCX]

**Supplementary Materials**

**Supplemental Table 1.** The function prediction of the three SNPs.

| SNP_ID | Chromosome | Position | Allele | TFBS | Splicing(ESE or ESS) |
| --- | --- | --- | --- | --- | --- |
| rs1853259 | 6 | 160067431 | A/G | Y | -- |
| rs7766006 | 6 | 160089248 | G/T | -- | Y |
| rs9457712 | 6 | 160066214 | A/G | Y | -- |

**Supplemental Table 2.** PCR primers and Unique Extend Primer (UEP) for MassArray genotyping.

| SNP_ID | 2nd-PCRP | 1st-PCRP | UEP_SEQ |
| --- | --- | --- | --- |
| rs1853259 | ACGTTGGATGCCCAAAGGAGTGTCTTGTAG | ACGTTGGATGTCCAGACCGATCTGATTCAC | GATTTATTAGGATAAGATTAAAGTACA |
| rs7766006 | ACGTTGGATGAAGTTGATCGCTGGGTCTAC | ACGTTGGATGGCTTTGCACAGACTCAAATC | CAGTTGGGCAACGCT |
| rs9457712 | ACGTTGGATGTACAGGAATGAACCACCACC | ACGTTGGATGGATACACTACTAGAGTACTG | CACCACTGGCCCCTC |
